# New Remedies of 1913

**Published:** 1914-07

**Authors:** 


### New Remedies of 1913

**Acid Atropinesulphuric**—Colorless prisms. **Sol.**: Easily in hot, difficultly in cold, water; also in diluted ammonia and diluted in acids; insoluble in organic solvents.—**Melt.**: 238° to 239° C.—**Uses**: To suppress salivation occurring during ether narcosis.

**Acitrin**—Ethyl Phenylcinchoninate. Yellowish, odorless, tasteless powder.—**Sol.**: Easily in organic solvents; difficultly in water.—Uric-acid Eliminant.—**Uses**: Gout, sciatica, and nerve disorders.—**Dose**: 0.5 to 1 Gm.

**Adigan**—A digitalis preparation which has been freed from digitonin and other saponin-like constituents by treatment with cholesterin. Said to be very effective while free from toxicity.

**Agobilin**—A remedy for biliary calculi marketed in the form of tablets each containing 0.088 Gm. strontium cholate, 0.032 Gm. strontium salicylate, and 0.04 Gm. phenolphthalein diacetate.—**Dose**: 2 tablets morning and evening.

**Aguma**—A nutrient preparation made from the soja bean.

**Aluminum Formaldehyde Silicate**—See Dreiaform.

**Aminoacetparaphenetidincaffeine Hydro-bromide**—See Kephalidon.

**Amylene Hydrate Isovalerate**—See Valamin.

**Anovarhyreoidin**—A serum from the blood of sheep from which the thyroid glands and ovaries have been removed. **Uses**: Osteomalacia, rachitis, etc.—**Dose**: Subcutaneously, 5 to 10 C.C. at intervals of 3 to 5 days.

**Antiluetin**—Potassium-Ammonium Antimonyl Bitartrate.—Antisyphilitic.—**Dose**: 1 to 2 C.C. of a 2.5% solution, subcutaneously. For man **the dosis therapeutica Sterilisans magna** is stated to be 0.75 Gm.

**Antimalazin**—A serum from ovariectomized sheep, and intended for use in Osteomalacia.

**Argulan**—Mercury Dimethylphenylpyrazolon.—46.8% Hg.—Antisyphilitic.

**Asthmolysin**—A sterile, aqueous solution of suprarenal extract from the infundibular portions of the hypophyses.—**Uses**: In asthma.—**Dose**: by subcutaneous injection, 1.1 C.C. (as marketed in ampuls. equals 0.0008 Gm. hypophysis extract.)

**Bismethylaminotetraminoarsenobenzene**—Sulphur-yellow powder; 26.5% As.—**Sol.**: Slowly but completely in water.—Antisyphilitic, like salvarsan.—**Dose**: 0.25 Gm. by injection.

**Borcholin**—See Enzytol.

**Bornyl Isovalerylglycolate**—See New-Bornyval.

**Brophenin** — Paraphenetidin Bromisovalerylaminoacetate. — White, almost odorless and tasteless powder.—**Sol.**: Very slightly in water.—**Melt**: 157° C.—Febrifuge and Analgesic.—**Uses**: Neuralgia, headache, lancinating pains in tabes, and in pains due to inflammatory conditions.—**Dose**: 0.5 to 1.5 Gm.

**Calcium Acetylparacresotinate**—See Ervasin-Calcium.

**Calcium-Casein**—See Larosan.

**Calcium Chloride-Gelatin**—See Kalzine.

**Calotropis Procera**—Mudar; Madar.—An evergreen shrub, the leaves and stems of which yield a milky juice which contains a substance having a digitalis-like action.

**Casein-Calcium**—See Larosan.

**Caviblen Pencils**—See Uranoblen.

**Chelinidin**—A turtle-tuberculin made like Koch's old-tuberculin.—A blackish-brown thick liquid.—**Uses**: For cure and immunization of tuberculous subjects.

**Chelinisal**—A suspension in physiological salt solution, of turtle bacilli rendered avirulent by repeated passage through animals. The preparation is injected intramuscularly for the cure and immunizing of tuberculous subjects.

**Choleval**—A 2% solution of collodial silver with 7.5% sodium glycocholate added as a protective colloid.—**Uses**: As urethral injection in gonorrhea.

**Choline Borate**—See Enzytol.

**Coagulin**—Thrombozyme from animal blood platelets.—**Sol.**: Alcohol, chloroform, water and physiological salt solution. **Uses**: To check hemorrhage.

**Coagulose**—A blood coagulant obtained by precipitating horse serum.—**Uses**: Hemophilia, hemorrhage, etc.

**Coeliacin**—An extract of the mesenteric glands, and marketed in tablets of 0.3 Gm. each (equals 0.3 Gm. fresh gland).—**Uses**: Scleroderma.—**Dose**: 0.3 Gm. once or twice daily.

**Colloidal Arsenic Trisulphide**—See Thiarsol.

**Colloidal Cupric Hydroxide**—See Cuprase.

**Colloidal Iron**—See Electromartiol.

**Colloidal Palladium Hydroxide**—See Leptynol.

**Colloidal Rhodium A.**—See Lantol.

**Colloidal Selenium**—See Electroselenium.

**Colloidal Sulphur**—See Sulfidal.

**Copper Hydroxide, Colloidal**—See Cuprase.

**Cordalen**—A digitoxin preparation each C.C. of which contains 0.00033 Gm. of the substance.—**Dose**: 1 C.C. subcutaneously.

**Cuprase**—Colloidal cupric hydroxide, marketed in ampuls of 5 C.C. each, and intended for subcutaneous injection in cancer.

**Cymarin**—New Apocynamarin.—Crystaline principle from Apocynum canabinum.—Colorless prisms.—**Sol.**: Difficultly in cold water, but easily in hot water and the usual organic solvents.—Diuretic.—**Uses**: As of digitalis.—**Dose**: 0.3 Mgm. (in tablet form); also intravenously in solution, 0.025 to 0.1 Mgm.

**Digimorval**—A digitalis, morphine, and valerian preparation intended for use in cardiac and circulatory disturbances, restlessness, insomnia, pains, etc., as well as coughs, pain in the chest, bronchitis, pleuritis, intestinal pains, and cholelithiasis. Marketed in tablets of 0.005 Gm. morphine, 0.05 Gm. powdered digitalis, and 3 drops menthol valerate, each.

**Dihydrocodeine**—See Paracodin.

**Dioxyanthroquinone**—See Istizin.

**Doramad**—Thorium-X for internal use as waters and injections in pernicious anemia, leucemia, angry ulcers, cancer, etc.

**Doriform**—Tetrabromopyrocatechol Bismuth. — Greenish-yellow, odorless powder.—**Uses**: As of iodoform, in nasal cases, otitis metia, tuberculous glands, etc.—Applied in oily or glycerinic 0.05% emulsion as spray or paint.

**Dreiaform**—Formaldehyde Aluminum Silicate.—Fine white powder.—**Uses**: As dusting powder on wounds, to render these sterile.

**Elarson**—Strontium behenolate, with chlorine and arsenic in peculiar combination.—Almost colorless, tasteless powder.—**Sol.**: Difficulty in alcohol, ether, and olive oil, insoluble in water.—**Uses**: Anemia, chlorosis, chorea, Basedow's disease, tuberculosis.—**Dose**: 1 tablet (equal to 0.0005 Gm. As equal to 1 drop Fowler's solution.)

**Electromartiol**—Colloidal iron obtained by electrolysis.—Marketed in granules, and in solution in ampuls.

**Electroselenium**—A colloidal selenium in the form of minute, coral-red grains obtained by electrolysis.—**Uses**: In cancer, by hypodermic or intravenous injection of a 2:100000 suspension.

**Enzytol**—Borcholin.—A choline borate which exerts an action in the system similar to that of the therapeutically active rays.—**Uses**: As bactericide in tuberculosis.—**Dose**: Intravenously, 0.01 to 0.25 Gm. in 10% solution.

**Ervasin-Calcium**—Calcium Acetylparacresotinate.—**Uses**: As of ervasin.

**Ethyl Phenylcinchoninate**—See Acitrin.

**Extractum Ovariale**—See Glanduovin.

**Folliculin**—A fluid extract of Tinnevelly senna pods, 1 Gm. representing 1 Gm. of the pods.—**Dose**: 1 to 3 teaspoonfuls per day.

**Formaldehyde Aluminum Silicate**—See Dreiaform.

**Galyl**—''1116''; Tetroxydiphosphaminodiarsenobenzene.—Yellowish to grayish-yellow powder.—Antisyphilitic.—**Uses**: Syphilis, trypanosomiasis, etc.—**Dose**: By intramuscular injection of an oily suspension of 0.3 Gm. per C.C., two injections of 0.5 C.C. each being made into the lumbar region on either side. Intravenous injections may also be given using an aqueous solution of 0.45 to 0.65 Gm.

**Glanduitrin**—A hypophysis extract, each C.C. of which contains the extractive matter of 0.2 Gm .of the infundibular portion of fresh hypophysis.—**Uses**: Subcutaneously and intramuscularly in inadequate labor pains.

**Glanduovin**: Extractum Ovariale.—An albumen-free ovarial extract.—Clear, light-yellow liquid, supplied in ampuls. 1.1 C.C. of the liquid equals 1 Gm. ovarial substance.—**Dose**: 2.2 C.C. subcutaneously.

**Glycerol Monoiodohydrin**—See Iodosapol.

**Glykobrom**—White, amorphous tasteless powder.—**Sol.**: Easily in ether and chloroform, difficultly in alcohol, and insoluble in water.—**Uses**: Where a prolonged bromine effect is desired.

**Grotan**—Sodium Parachlormetacresol. — Disinfectant.—Marketed in tablets of 1 Gm. each and rapidly soluble in water. Used in 0.5% solution.

**Hemanthine**—An alkaloid from Haemanthus toxicarius, and closely related, chemically and therapeutically, to the tropeine series.

**Hygralon**—A mercury-potash soap containing 30% Hg. and intended for the "colorless" inunction treatment.

**Hypophysin**—A 1:1000 solution of the sulphate of an active base obtained from the hypophysis.—**Uses**: Intramuscularly to promote uterine contractions.

**Igebin**—Tablets said to consist chiefly of "dimethylaminophenyldimethylpyrazolon with a cinchona alkaloid and the active principle of Kola nut."—**Uses**: Influenza, colds, fever, neuralgia, headache, and articular rheumatism, and to prevent seasickness.

**Iodosapol**—Glycerol Monoiodohydrin.—A guaiacol compound used as an antiseptic dressing and for disinfecting the hands and surgical instruments, either undiluted or in 1:4 solution.

**Iodtriferrin**—Iron Paranucleinate.—Reddish-brown powder.—**Sol.**: Easily in diluted alkalies and concentrated acids; insoluble in water and diluted acids.—**Uses**: Anemia, scrofula, etc.—**Dose**: 0.2 Gm.

**Iron Paranucleinate**—See Iodtriferrin.

**Istizin**—Dioxyanthraquinone.—Gold or orange-yellow leaflets, or orange-yellow powder.—**Sol.**: Difficultly in usual organic solvents, but more easily in hot glacial acetic acid.—Cathartic.—**Dose**: 0.1 to 0.5 Gm.; usual average dose 0.3 Gm.

**Kalzine**—Calcium chloride-gelatin, containing 5% calcium chloride and 10% gelatin.—**Uses**: subcutaneously in hemorrhagic diathesis, hemorrhage of the internal organs, recurrent exudative pleuritis, Basedow's disease, and bronchial asthma.

**Kephalidon** — Aminoacetparaphenetidinecaffeine Hydrobromide.—White, bitter, crystalline powder.—**Sol.**: Slowly in cold, more easily in hot water, and in alcohol.—**Melt.**: 192° C.—**Uses**: Migraine, neurasthenia, lancinating pains and headaches.—**Dose**: 0.3 Gm. single; 1.5 Gm. per day.

**Kinazyme**—An active extract of spleen with the pancreatic enzymes.—**Uses**: To increase digestion, nutrition, and leucocytosis.

**Kresophine**—A coal-tar derivative.—Reddish-brown liquid, easily mixable with all organic solvents.—**Uses**: As of other coal-tar products.

**Lantol**—Colloidal Dhodium A.—Marketed in 1:1000 solution in ampuls, and in keratinized capsules, and intended for use in cancer and septic diseases.

**Laorsan**—Casein-Calcium.—Fine, white, tasteless powder.—**Sol.**: Hot milk.

**Laudanon**—Combination of various opium alkaloids, and intended for use instead of opium. Marketed in ampuls of 1 C.C. each, and containing:

| | Laudanon I. | Laudanon II. |
|---|---|---|
| Morphine | 0.01 Gm. | 0.01 Gm. |
| Codeine | 0.001 Gm. | 0.001 Gm. |
| Papaverine | 0.002 Gm. | 0.0001 Gm. |
| Thebaine | 0.0005 Gm. | 0.0005 Gm. |
| Narceine | 0.0005 Gm. | 0.0001 Gm. |
| Narcotine | | 0.001 Gm. |

**Leptynol**—Colloidal Palladous Hydroxide.—Described as "a colloidal solution of wool fat-palladous hydroxide in liquid paraffin, containing 2.5% Pd as Pd $(OH)_2$; 1 C.C. equals 25 Mgm. Pd."—**Uses**: In obesity.—**Dose**: 2 C.C. deeply injected into abdominal fat.

**Leukozon**—A mixture of approximately equal parts calcium perborate and talcum.—**Uses**: Disinfectant dusting powder either pure or mixed with other remedies.

**Ludyl** — "1151"; **Phenyldisulphaminotetroxydiaminoarsenobenzene.**—Yellowish or grayish yellow powder.—**Uses** and **Doses**: As of Galyl (which see).

**Madar**—See Caloptropis Procera.

**Menthol Acetylsalicylate**—See Menthospirin.

**Menthospirin—Menthol Acetylsalicylate.**—Viscid, light-yellow liquid, marketed in pearls of 0.25 Gm. each.—**Uses**: Acute laryngitis, frontal headache, earache, coryza, etc.—**Dose**: 2 to 3 pearls 2 to 3 times daily.

**Mercury Dimethylphenylpyrazolon**—See Argulan.

**Merlusan**—A mercury-albumen compound.—**Sol.**: Alkalies; insoluble in acids.—Antisyphilitic.—**Uses**: Syphilis, and particularly in gonorrhea in alteration with other remedies.

**Metarsan**—An organic arsenic preparation intended for subcutaneous use instead of salvarson or neosalvarsan in contagious pleuro-pneumonia in horses.

**Mudar**—See Calotropis Procera.

**Narkodeon**—Pastilles each of which contains 0.001 Gm. narcotine hydrochloride and 0.005 Gm. codein hydrochloride, with tolu and compound acacia powder. Used in catarrh of the respiratory passages.

**Neolysol**—A fluid, odorless chlorcresol preparation.—**Uses**: Antiseptic and deodorant like lysol.

**Neurokardin**—A solution of the therapeutically active resins from the rhizome of one of the piperaceae.—**Uses**: Nervous headaches, neurasthenia, hysteria, heart diseases, arteriosclerosis.

**New Apocynamarin**—See Cymarin.

**New-Bornyval**—Bornyl Isovalerylglycolate.—Colorless, almost odorless and tasteless liquid.—**Sol.**: Easily in alcohol, ether, benzene, and oils; insoluble in water.—Sedative and Nervine. **Uses**: Excitement of all kinds, in debility and collapse, neuroses, and disturbances of the circulatory digestive, and central nervous system.—**Dose**: 1 to 3 pearls (each 0.25 Gm.) 2 to 4 times daily.

**Novocolchinin**—A mixture of 60 parts quinine sulphate and 40 parts novocol (sodium guaiacolphosphate).—**Sol.**: Easily in water and milk.—**Uses**: Whooping cough in children. **Dose**: 1 to 3 tablets (in which form marketed).

"1116"—See Galyl.

**Paracodin**—Dihydrocodeine—Marketed as hydrochloride and tartrate, both of which are used like codeine for checking coughs, etc.—**Dose**: 0.02 to 0.05 Gm.

**Paraiodorthosulpho-oxycylohexatrienpydridine.**—See Yatren.

**Paraphenetidin Bromisovalerylaminoacetate.**—See Brophenin.

**Perborax**—A compound of sodium polyborate and perborate. About 4% active oxygen.—**Uses**: As of sodium perborate

**Perhydrit**—A urea-perhydrol compound.—White powder.—**Sol.**: 2.5 water.—**Uses**: As of hydrogen peroxide.

**Phenyldisulphaminotetroxydiaminoarsenobenzene**—See Ludyl.

**Pinosol**—A tar product.—Yellow to brownish, honey-like liquid, of mild empyreumatic odor and taste at first oily and aromatic and finally bitterish and pungent.—**Misc.**: Fatty and volatile oils, fats, petrolatum, and wool fat; affords emulsion with dilute alkaline solutions; insoluble in water. **Uses**: Instead of tar and empyreumatic oil of birch.

**Polygonum Hydropiper**—Water Pepper.—Used in form of fluid extract in hemoptysis, and in vesical, gastric, and hemorrhoidal hemorrhage.—**Dose**: 30 to 40 drops three times daily.

**Potassium-Ammonium Antimonyl Bitartrate**—See Antiluetin.

**Rademanit**—A radium-charcoal preparation possessing a decided radioactivity.—**Uses**: Cancer.

**Resaldol**—Resorcinolbenzoylcarboxylic-acid Ethylester—Light yellow crystals, very slightly soluble in water.—**Melt.**: 134° C.—**Uses**: Dysentery.

**Resorcinolbenzoylcarboxylic-acid Ethylester**—See Resaldol.

**Rhodium A, Colloidal**—See. Lantol.

**Riopan**—A concentrated preparation made from Rio ipecac, and containing the hydrochlorides of the ipecac alkaloids to the extent of 50%. 1 Riopan equals 20 ipecac.—Brownish powder soluble in water.—Marketed in tablets (each 0.001 Gm.) ipecac alkaloids equal 0.015 Gm. ipecac.

**Romanxan**—A nutrient said to consist of the protalbumoses of milk albumen, metaphosphoric acid, and iron salts.—**Uses**: nutritional disturbances, convalescence from disease, etc.—**Dose**: 10 to 12 Gm. per day. May also be given in enema.

**"Stable" Scopolamine**—A scopolamine solution rendered stable by the addition of 10% mannit, and marketed in ampuls containing respectively 0.0003 Gm., 0.0005 Gm. and 0.001 Gm. Scopolamine hydrobromide per C.C.

**Secretogen**—A combination of the gastric (pyloric) and pancreatic (duodenal) secretions.—**Uses**: To control digestive insufficiencies.

**Sennatin**—A fluid preparation from senna leaves.—Dark, clear, stable liquid.—**Uses**: Constipation.—**Dose**: 1 to 3 Gm. internally; also given subcutaneously and intramuscularly.

**Sennax**—A purgative glucoside obtained from senna leaves, and marketed as a lactose trituration.—**Dose**: 1 table, or 4 C.C. solution (in which form supplied) each equals 0.75 Gm. glucoside.

**Skiargan**—A sterile collargol solution containing 9% Ag.

**Sodium Parfachlormetacresol**—See Grotan.

**Solargyl**—A compound of silver oxide with proteoses and their cleavage products.—Odorless leaflets of blue, metallic luster. **Sol.**: Easily in cold water and in glycerin; insoluble in absolute alcohol, ether, chloroform ,and carbon disulphide.

**Sulfidal**—Sulfoid; colloidal Sulphur.—Grayish white powder insoluble in the usual solvents.—**Uses**: As of sulphur externally, either in powder, or in 10% soaps, ointments, etc.

**Sulfoid**—See Sulfidal.

**Tenosin**—A combination of betamidoazolthylamine and paraoxyphenylethylamine, the active constituents of ergot.—Colorless, sterile liquid.—**Use**: Uterine tonic and styptic in obstetrics.—**Dose**: 1 C.C. of solution (as marketed).

**Terpacid**—Fenchone from fenchyl alcohol.—Colorless, mobile liquid; camphoraceous, bitter taste.—**Sol.**: Most organic solvents.—**Uses**: Gout, rheumatism, neuralgia.

**Tetrabromopyrecatechol Bismuth**—See Doriform.

**Tetroxydiphosphaminodiarsenobenzene**—See Galyl.

**Theoform**—Condensation product of Theobromine with substances splitting off formaldehyde.—white, very bitter powder.—**Sol.**: 50 water.—diuretic.—**Dose**: 1 Gm.

**Thiarsol**—Colloidal Arsenic Trisulphide.—Supplied in sterilized tubes and in solution.

**Trien**—See Yatren.

**Tryen**—See Yatren.

**Uranoblen**—A silver-uranine compound. 40% Ag.—Reddish-brown powder soluble in water.—Bactericide.—**Uses**: In gonorrhea, in the form of urethral bougies, named "caviblen pencils.

**Valamin**—Amylene Dydrate Isovalerate.—Colorless, neutral liquid; valerian odor and taste.—**Misc.**: All proportions with oils, very slightly soluble in water.—**Uses**: As sedative in all cases where valerian is indicated.—**Dose**: 0.25 Gm.; in mild nervous insomnia, 0.5 Gm.

**Water Pepper**—See Polygonum Hydropiper.

**Yatren**—Tryen, Trien; Paraiodorthosulpho-exycyclohexatrien. pyridine.—Yellow, odorless powder.—**Sol.**: Hot water.—Bactericide.—**Uses**: 10 to 20% solution, also as dusting powder for wounds.

**Yohydrol**—New name for yohimbine hydrochloride.

